# Anxious man with blurred vision

**DOI:** 10.11604/pamj.2015.20.361.5349

**Published:** 2015-04-14

**Authors:** Adil Belmekhtar, Daoudi Rajae

**Affiliations:** 1 Université Mohammed V Souissi, Service d'Ophtalmologie A de l'Hôpital des Spécialités, Centre Hospitalier Universitaire, Rabat, Maroc

**Keywords:** Eye, central serous chorioretinopathy, stress

## Image in medicine

A 34-year-old man with history of stress presented to the emergency department with blurred vision in the left eye for the past week. There has been no vision change in his right eye. The vision acuity of the left eye was 20/100. The fundus examination revealed a localized serous detachment of the macula (A). The fluorescein angiography (B) and the ocular coherence tomography (OCT) (C) confirmed the diagnosis of Central serous chorioretinopathy. Central serous chorioretinopathy (CSCR) is characterized by the accumulation of subretinal fluid at the posterior pole of the fundus, causing a retinal detachment. When the detachment spreads into the macula, patients typically describe decreased vision acuity, metamorphopsia and a scotoma. CSCR is predominant in caucasians men between 20 to 45 years of age, and is highly linked to stress and the use of corticosteroid. The diagnosis is based on the ophthalmoscopic findings and results of fluorescein angiography, which shows single or multiples leakage points. The ocular coherence tomography (oct) contribute in the diagnosis by demonstrating the neurosensory detachment and is an objective way of surveillance. Generally, CSCR has a good prognosis for spontaneous visual recovery. However, some patients develop chronic CSCR or subretinal fibrosis.

**Figure 1:**
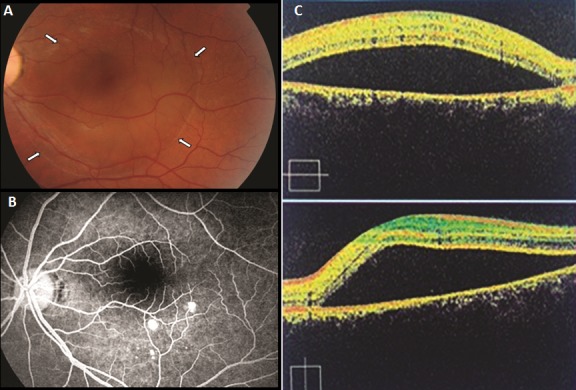
(A): photography of fundus showing CSCR limites; (white arrow) (B): the fluoresceine angio showing leakage points; (C): OCT scan demonstrating the neurosensory detachment

